# Shapes and Patterns of Heme-Binding Motifs in Mammalian Heme-Binding Proteins

**DOI:** 10.3390/biom13071031

**Published:** 2023-06-23

**Authors:** Dhruv C. Rathod, Sonali M. Vaidya, Marie-T. Hopp, Toni Kühl, Diana Imhof

**Affiliations:** 1 Pharmaceutical Biochemistry and Bioanalytics, Pharmaceutical Institute, University of Bonn, D-53121 Bonn, Germany; 2 Department of Chemistry, Institute for Integrated Natural Sciences, University of Koblenz, D-56070 Koblenz, Germany

**Keywords:** heme-binding protein, heme-binding motif, CP motif, structural patterns

## Abstract

Heme is a double-edged sword. On the one hand, it has a pivotal role as a prosthetic group of hemoproteins in many biological processes ranging from oxygen transport and storage to miRNA processing. On the other hand, heme can transiently associate with proteins, thereby regulating biochemical pathways. During hemolysis, excess heme, which is released into the plasma, can bind to proteins and regulate their activity and function. The role of heme in these processes is under-investigated, with one problem being the lack of knowledge concerning recognition mechanisms for the initial association of heme with the target protein and the formation of the resulting complex. A specific heme-binding sequence motif is a prerequisite for such complex formation. Although numerous short signature sequences indicating a particular protein function are known, a comprehensive analysis of the heme-binding motifs (HBMs) which have been identified in proteins, concerning specific patterns and structural peculiarities, is missing. In this report, we focus on the evaluation of known mammalian heme-regulated proteins concerning specific recognition and structural patterns in their HBMs. The Cys-Pro dipeptide motifs are particularly emphasized because of their more frequent occurrence. This analysis presents a comparative insight into the sequence and structural anomalies observed during transient heme binding, and consequently, in the regulation of the relevant protein.

## 1. Introduction

**Figure 1:**
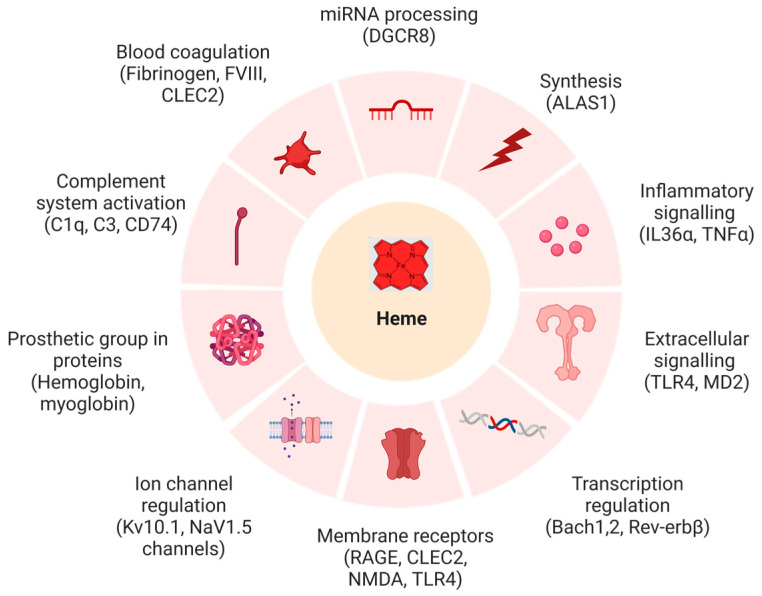
Biochemical systems and targets affected by heme with example proteins (in brackets), whose functions were shown to be impaired upon heme binding (created with BioRender (© 2023)). Abbrevations: ALAS1: δ-aminolevulinic acid synthase 1, Bach-1: BTB domain and CNC homolog 1, Bach-2: BTB domain and CNC homolog 2, CLEC2: C-type lectin-like type II transmembrane receptor, C1q: complement component 1q, C3: complement component 3, CD74: cluster of differentiation 74, DGCR8: DiGeorge critical region 8, FVIII: anti-hemophilic factor, IL-36α: interleukin-36α, Kv10.1, MD2: myeloid differentiation factor 2, NMDA: *N*-methyl-D-aspartate, Nav1.5: sodium channel protein type 5, RAGE: receptor for advanced glycation end products, Rev-erbB: nuclear receptor subfamily 1 group D, TLR4: Toll-like receptor 4, TNFα: tumor necrosis factor α.

Heme, also called iron protoporphyrin IX, is an essential multifaceted molecule that has distinct functions in both plants and animals [1,2,3]. In addition to being a prosthetic group of many proteins (hemoglobin, cytochromes, etc.), it plays a pivotal role as an effector molecule in regulatory and signaling mechanisms in living organisms (Figure 1) [2]. This functional characteristic of heme is due to its interactions with proteins involved in various physiological events in a transient fashion [4,5]. The heme–protein interactions take place because of a distinct motif, referred to as the ‘heme-regulatory motif’ (HRM) or the ‘heme-binding motif’ (HBM). Heme binding to HRMs can regulate the protein, although heme association with HBMs does not necessarily impair a protein’s function [6,7,8,9]. In general, such motifs can be defined as short amino acid sequences that contain at least one heme-coordination site on the surface of the protein [7,8]. Several studies have identified and characterized motifs containing a cysteine–proline dipeptide, commonly known as a CP-motif, as well as motifs containing histidine and tyrosine (H/Y) as the coordinating residues involved in transient heme binding [6,9,10].

Analysis of the mammalian HRM-containing proteins described so far (Table S1) revealed that coordination to cysteine seems to play a major role in transient heme-binding events [6,8,11,12]. In some proteins, cysteine alone is sufficient for heme coordination when occurring in an appropriate sequence context [13]. Additionally, a Cys-Ser (CS) motif was found in the HRM of stanniocalcin-1 [14]; however, descriptions of the aforementioned CP-motifs are far more abundant (i.e., 31% of the proteins summarized in Table S1). In previous studies, we provided data and in-depth structural analyses for different classes of Cys-based peptides binding to Fe(III)-heme, focusing on the elucidation of deviations between motifs containing only one (C/CP) or additional iron-ion-coordinating amino acids (i.e., H and/or Y) [5,6,8,9]. However, the CP motif was not further considered with respect to contributions from the CP environment, although one important finding was the predominant penta-coordination of CP-containing sequences irrespective of the occurrence of a further His or Tyr within the sequences. In 2011, Li et al. observed an increase in the incidence of CP motifs in a non-redundant dataset of 125 heme proteins [15], although with weaker signature than the canonical, covalently bound CXXCH motif [16]. It was suggested that proline supports the coordination of the cysteine (in the thiolate form) to the Fe(III)-heme complex [17,18]. The proline residue in these structures was found to introduce a bend in the backbone preventing them from contact with the heme face. We confirmed this finding through structural analysis of the CP motif within dipeptidyl peptidase 8 and functional analysis of the catalytic activity of the full-length protein [6,19].

In the context of transient heme–protein interactions, it is imperative to understand the molecular and chemical bases of these interactions, which are conferred by a coordinative bond between the central iron ion and a heteroatom in the side chain of the coordinating residue, hydrophobic interactions, and the π–π stacking of adjacent amino acids with the porphyrin ring, as well as electrostatic interactions and hydrogen bonding with the propionate side chains of the porphyrin ring. Thus, the coordinating amino acid, as well as its environment, influence heme binding to a protein [5]. In addition to different spectroscopic methods used to investigate heme binding to proteins, which include techniques such as UV/vis, *r*Raman, *cw*EPR, and 2D-NMR spectroscopy [5,8,11], computational tools such as HeMoQuest complement these methods in predicting transient heme associations with distinct proteins [12,20,21].

We herein report on peptides primarily derived from known heme-binding proteins to further validate the proposed critical role of the CP motif for heme–protein interactions in comparison to H/Y-based motifs. This analysis is supported by the structural examination of these motifs and the evaluation of patterns and positions of the heme-interacting residues. Therefore, we intend to broaden the understanding of the basis of transient heme binding, considering similarities in the structural patterns of conserved regions within the respective proteins. This approach allows us to derive general consensus sequences of the respective HBMs/HRMs depending on the coordination site.

## 2. Materials and Methods

### 2.1. Peptide Synthesis, Purification, and Analytics

A standard Fmoc (N-(9-fluorenyl) methoxycarbonyl) protocol was applied for the automated solid-phase peptide synthesis of peptides **11**–**17** using an EPS 221 peptide synthesizer (Intavis Bioanalytical Instruments AG, Cologne, Germany), as previously described [5,6,8,13]. Peptides were synthesized as amides using Rink amide MBHA resin (0.53 mmol/g) as the solid phase, while HBTU and HOBt were utilized as coupling agents. Peptide cleavage was performed by applying 100 µL/100 mg resin reagent K and 1 mL/100 mg 95% TFA on ice. Crude products were purified using semi-preparative HPLC with a Knauer Eurospher 100 column (C18, 250 × 32 mm, 5 μm particle size, 100 Å pore size) on a Shimadzu LC-8A system. Analytical HPLC served to verify peptide purity and was performed on a Shimadzu LC-10AT system with a Vydac 218TP column (C18, 4.6 × 25 mm, 5 μm particle size, 300 Å pore size). Gradient elution was performed using the following solvent system: eluent A: 0.1% TFA in water; eluent B: 0.1% TFA in acetonitrile + 0.1 % TFA. Mass spectrometry analysis using LC-ESI MS on a micrOTOF-Q III device (Bruker Daltonics GmbH, Bremen, Germany) connected to a Dionex UltiMate 3000 LC (Thermo Scientific, Waltham, MA, USA) served to confirm peptide identity. Elution was achieved using an EC 100/2 Nucleoshell RP18 column (C18 Reversed Phase, 100 × 2 mm, 2.7 μm particle size, 90 Å pore size) with water and acetonitrile (each containing 0.1% acetic acid) as the solvents. Information concerning the analytical data of the peptides is presented in the Supporting Information (Table S2). For peptide content determination, the peptides were hydrolyzed using 6 N HCl at 110 °C for 24 h and subsequently prepared for analysis on an LC 3000 system (Eppendorf-Biotronik, Berlin, Germany).

### 2.2. Analysis of Heme-Binding Peptides by UV/vis Spectroscopy

Heme binding to protein-derived peptides and controls (Table 1) was investigated by UV/vis spectroscopy, as described previously [5,6,8,13]. Briefly, the peptides (constant concentration: 20 µM) were incubated for 30 min with varying concentrations of heme (0.4–40 μM) in 100 mM HEPES buffer (pH 7.0). Absorbance spectra were recorded on a Multiskan GO spectrophotometer (ThermoScientific, Dreieich, Germany) in the range of 300–600 nm. Difference spectra were generated by calculating the difference in the absorbance of pure heme and peptide and the absorbance of the peptide–heme complex. Dissociation constants (K_D_) were determined using GraphPad prism 9.3.1 software and the previously established equation from Pîrnău and Bogdan [13,22,23,24].

### 2.3. Structural Analysis

A comprehensive list of heme-binding proteins (HBPs) was prepared from the available literature reports (Table S1). To transpose the knowledge acquired from the sequence-based studies to structural patterns, 3D structure analysis of the proteins was required. Each protein was therefore queried on the Universal Protein Knowledgebase (UniProtKB) with the filter for mammalian proteins (e.g., *Homo sapiens*, *Mus musculus*, and *Rattus norvegicus*) [25]. For the 3D structures, the RCSB database (RCSB-PDB) was employed, from which crystal structure analyses of each protein were searched and downloaded where available [25,26]. For those proteins for which crystal structures were not available, the respective AlphaFold structure (marked with ** in Table S1) was used for further analysis [27]. The nonapeptide sequences of each HBM were grouped herein into four primary classes, i.e., CP-, C-, H-, and Y-based motifs [15]. An analysis was performed where each HBM was visualized along with its side chains on UCSF Chimera (version 1.16) [28]. Information, such as the location of the coordination residues (C, H, and/or Y), was obtained, along with its hydrogen bonding patterns. Here, the location implies the position of the coordinating residue on the secondary structure in the folded protein. From this information, patterns were observed and analyzed for structural similarities or differences upon superimposition of the HBMs, separately for each class. Figures were prepared using UCSF Chimera (version 1.16) and BioRender (© 2023) [26].

## 3. Results

### 3.1. Experimental Results from UV/vis Studies

**Figure 2:**
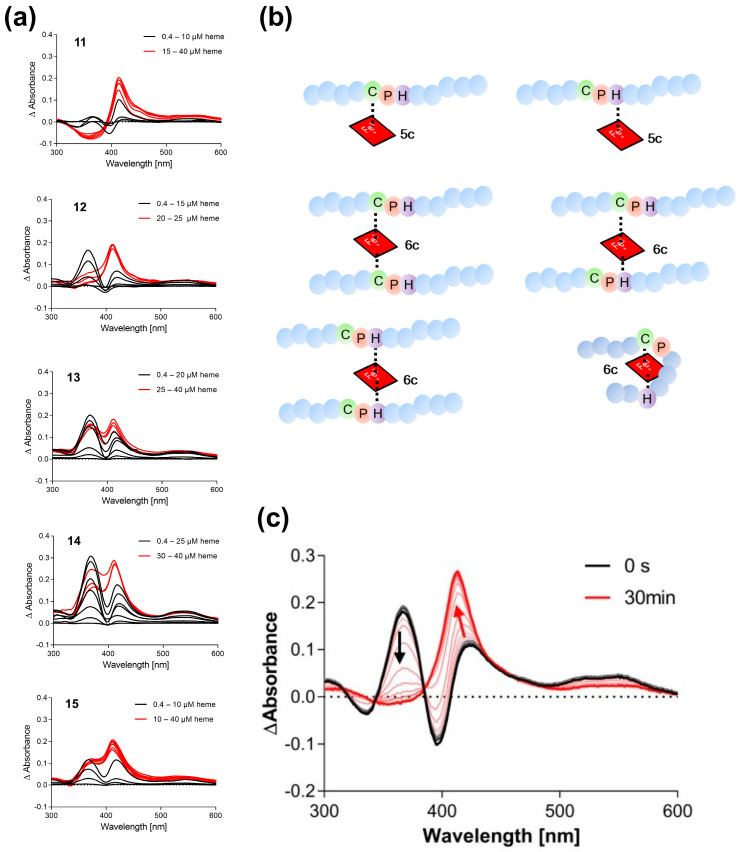
UV/vis binding studies on Cys-based peptide–heme complexes. (**a**) Heme titration of peptides **11**–**15**. Different binding modes occur depending on the position of the second coordination site (H/Y) after incubation for 30 min. (**b**) Scheme of possible peptide–heme complexes based on the sequence features of CP-peptides **11**–**15**. The distance of at least three amino acids between the two coordination sites is needed for loop formation to take place. (**c**) Time-dependent (0–30 min) complex formation of peptide **11** with heme (20 µM, ratio 1:1).

The CP-containing peptides considered in this study are summarized in Table 1. Peptides **1**–**10** were derived from known HBPs (Table S1) [5,6,8,13,16], such as iron regulatory protein 2 (IRP2) [29,30,31], heme-regulated eIF2α kinase (HRI) [32,33,34], and DiGeorge critical region 8 protein (DGCR8) [32,35], as well as recent reports of potential new HBPs (Table S1) [29,30,31,32,33,34,35,36,37,38,39,40,41,42,43,44,45,46,47,48,49,50,51,52,53,54,55,56,57,58,59,60,61,62,63,64,65,66,67,68,69,70,71,72,73,74,75,76,77,78,79,80,81,82,83,84,85,86,87,88,89,90,91,92,93,94,95,96,97,98,99,100,101,102,103,104,105,106,107,108,109,110,111,112,113,114,115,116] or represent controls; peptides **11**–**17** were added to provide evidence about the minimal distance between two coordination sites, i.e., cysteine and histidine, to enable loop-like hexa-coordination (Figure 2). Peptide **11** is a synthetic peptide, derived from IRP2. Peptides **12**–**15** are mutants of peptide **11**, changing the position of His as the coordinating residue from +1 to +5 residues at the C-terminal of proline. This issue was raised upon prior studies on Cys-based peptides [8,12,15], in which it was suggested that a minimal spacer length of 2–3 amino acids between the coordination sites is required. All peptides were pre-screened for their heme-binding capacity by UV/vis spectroscopy with an established experimental setup (Table 1, Figure 2) [6,8,11,13]. The UV/vis experiments revealed interesting insights into the coordination states of the individual CP-peptides (Figure 2). In particular, CP(H)-peptides **11**–**15** displayed different coordination states depending on the distance of CP and H. According to the UV/vis spectra, various peptide–heme complexes were present in a highly concentration-dependent manner (Figure 2a). A band shift to ~370 nm is characteristic for penta-coordinated (5c) complexes, typically observed for CP motifs; a shift to ~420 nm mostly represents a hexa-coordinated complex (6c) of different complex architecture or a penta-coordinated complex having histidine as the coordination site (Figure 2b).

It appears that the closer the histidine residue is located to the CP motif (**11**, **12** compared with **13**–**15**), the higher the tendency to form a hexa-coordinated complex (shift to ~420 nm). Monitoring the complex formation of peptide **11** with heme (ratio 1:1) suggests that heme binding to the CP motif (~367 nm) occurs faster, but eventually the heme moiety is transferred to the histidine residue as can be seen from the change in the band shift from ~370 nm to ~420 nm (Figure 2c). In contrast, maxima at ~370 nm and ~420 nm are observed in the case of peptides with a distance <2 residues between CP and H (**13**–**15**) indicating the simultaneous presence of different complexes. Inverse CP motifs as in **16** and **17** interact with heme but showed deviating binding behavior compared with wild-type peptide **7** that exhibited a characteristic shift to ~370 nm (Table 1, Figure S1).

### 3.2. Structure Evaluation Using Computational Tools

**Figure 3:**
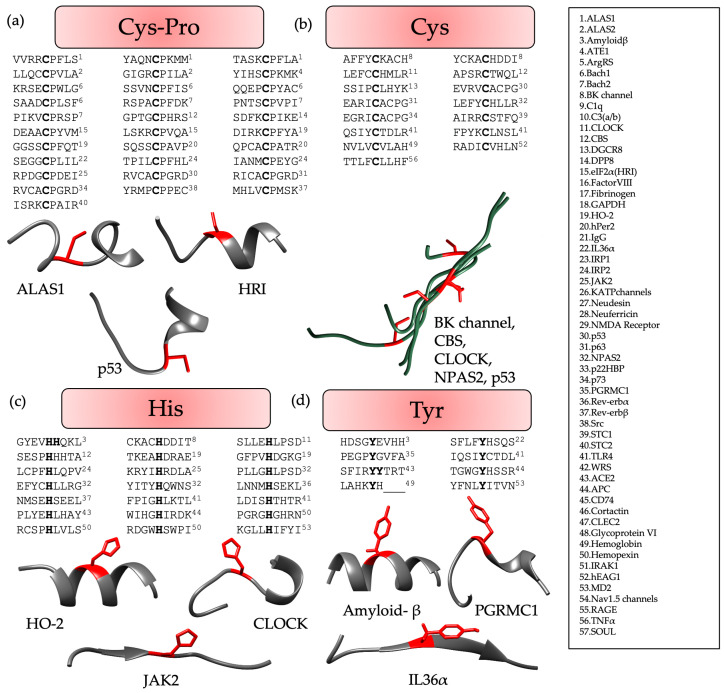
Evaluation of structures available from X-ray crystallography or AlphaFold (Table S1) for (**a**) CP motifs, (**b**) C-based motifs, (**c**) H-based motifs, and (**d**) Y-based motifs. The coordinating residues, i.e., Cys (**a**,**b**), His (**c**), and Tyr (**d**) are highlighted in red. Each figure displays selected examples of representative protein sequences (from the N-terminus (left) to the C-terminus (right)) either individually (**a**,**c**,**d**) to highlight the differences in the conformations or as a superimposition (**b**), in case no pronounced structural features were observed. Here, ‘_’ represents the residues that were not contained in the given PDB structure because of their position at the terminus. HBMs of the proteins highlighted in “grey” are not identified. Additional information concerning the conformation observed for each HBM can be found in Table S1.

Analysis of the structures was conducted to identify distinct patterns occurring in the CP-containing proteins disclosed exciting insights. Therefore, the 3D structures available for the proteins (Table S1) were examined in more detail, revealing that the CP motif was mostly found in the loop that joined two α-helices (Figure 3a). Apart from this, there were also a few examples of CP motifs in loops between an α-helix and a β-sheet or two β-sheets. This positioning was also consistent in the AlphaFold structures that were considered for the proteins (Table S1). Herein, the CP motif with a per-residue confidence score (pLDDT) higher than 70, and thus signifying good quality of the predicted structure, was analyzed, and found to be in the loop joining two secondary structure elements, i.e., the α-helix and β-sheet. When analyzing the available crystal structure of p53 (PDB: 7XZZ) it was, however, realized that the cysteine was the C-terminal residue of the α-helix in front of the loop (P in the loop), which was then connected to the β-sheet. Similarly, analysis of the AlphaFold structure of IRP2 revealed it to be the same as cysteine is the C-terminal residue of the α-helix before the loop starts herein as well. Analysis of the CP motif containing proteins is displayed in Figure 3a.

Similar comparative analysis of HRMs with Cys-based motifs revealed that it was predominantly found within a flexible long-distance loop, without any distinct structural features (Figure 3b), i.e., no bend was observed, as found in case of the CP motifs [6,8,20].

In contrast to CP-based motifs, H- and Y-based HBMs were particularly found in the proteins in the center of an α-helix or a β-sheet. However, these patterns showed less significant contributions to heme binding compared with CP motifs, due to the higher flexibility of the loops harboring the CP motif, as compared with the compact secondary structures of an α-helix or a β-sheet. However, H- and Y-based motifs did not show any structural pattern, but are rather inconsistent in their conformations (Figure 3c,d). All the sequences analyzed lay within the consensus sequences derived earlier [10].

The analysis of the residues surrounding the coordination site(s) was again more pronounced for CP than for the other motifs. Examining heme-binding CP motifs revealed that the N-terminal included at least one, but primarily two, aliphatic hydrophobic residues (~87%), such as A (40%), I (~27%), V (~23%), and L (~17%), with the latter amino acids—if grouped—being present in 50% of the CP-containing proteins (Table S1). It was also observed that these aliphatic hydrophobic residues are, in most cases, combined with one or two polar residues, with S, T, or Q found in ~66% of the HBPs and D, E, R, or K contributing to ~23% of the respective proteins (Table S1). The cysteine residue is located at the beginning of the loop; therefore, the residues behind the proline are placed within the loop. Herein, a more variable composition of the amino acids can be observed, however, with a higher frequency of aromatic amino acids (F, Y, W) placed close to the bend-inducing proline residue (in 40% of the proteins).

For C-based motifs derived from HBPs (Table S1), we observed that ~46% have 1–3 aromatic residues (F, Y, and W) and ~93% have either aromatic and/or aliphatic hydrophobic residues (I, L, and V) at the N-terminus. Again, many of the described HBMs (~60%) possess a combination of these hydrophobic residues with polar residues (S > E > T, N, Q, and D), but still approximately 80% of the C-based HBPs (Table S1) exhibit more hydrophobic aliphatic or aromatic amino acids over polar ones. The distribution of these amino acids in the proteins, particularly at the N-terminal four residues of the motif, is also reflected by the peptides studied herein and previously [5,6,8,13].

Regarding H- and Y-based motifs, the situation is hampered by the fact that for Y-based motifs, only eight mammalian HBPs have been described so far (Table S1). We thus focused our analysis on the H-based HRMs only. Therefore, it was observed that ~90% of the HBPs have one or two hydrophobic residues, including L, V, and I, combined with an aromatic amino acid (F, Y, and W). Moreover, 62% of the HRMs contain at least two aliphatic residues (e.g., LL, LI) or one aliphatic and one aromatic residue (e.g., YV, LF, LY, or YI). In 62% of these proteins, these amino acids are combined with polar residues, such as S or E. This pattern is also reflected in the H-based peptides with aliphatic residues (L/A > V > I) being preferred over aromatic residues; however, for polar residues, a higher occurrence of Q is found.

## 4. Discussion

The results of the present study demonstrate that the existence and the sequence position of additional coordination sites for heme in HBPs have a major impact on the binding mode, confirming prior results [6,11]. It was observed that recruitment via a CP motif and subsequent heme transfer to an additional residue, such as histidine, can occur. This effect was observed to depend on the incubation time and the peptide–heme ratio. The identification and prediction of putative heme-binding sites is conditional to the understanding of the sequential and structural patterns assisting heme binding. Even though many classification schemes [5,10] have been developed based on the protein/peptide sequence [18], structural signatures occurring in HBMs/HRMs have not yet been mapped. This study provides a basis for such a classification scheme to be developed in the future through analysis of the known transiently heme-binding proteins of mammalian origin.

The analysis of each type of motif, i.e., CP-, C-, H-, or Y-based, revealed that CP motifs are predominantly found in the loop region joining two secondary structures. Hence, this provides more flexibility for heme binding than the other iron-coordinating residues present directly within a secondary structure element. From the proteins available so far (Table S1), it can be concluded that C-, H-, and Y-based motifs did not show any significant structural pattern, but different possibilities exist. However, some sequential patterns, such as the presence of particular amino acids at the N-terminus, were found for these three types of motifs. To the best of our knowledge, no detailed analysis of HRM-containing proteins has been performed that primarily focuses on the structural requirements of HRMs of all different classes of HRM motifs, i.e., CP-, C-, H-, and Y-based motifs. Although our study only indicates a similar fold within different proteins for CP motifs, and thus, a structural pattern, the majority of protein candidates of the other classes have been identified after the CP-motifs were reported as HRMs. Thus, an increase in the number of further examples for the other motifs can be expected. Future exploration of these heme-binding proteins may provide a significant structural pattern supporting heme association.

In addition, there were other factors hindering the analysis of such structural patterns, one of which was the availability of 3D structures. For approximately 50% of the studied proteins, crystal structure analyses are still missing, and some available crystal structures do not contain the HBMs/HRMs [29,31,35,92,93]. In such situations, AlphaFold may support the study by providing select structural models of some of the proteins. However, in the majority of the AlphaFold structures, the heme-binding regions demonstrated very poor accuracy scores (pLDDT < 50). Hence, analysis from these structures is not completely reliable. Among the 56 proteins (Table S1) which were analyzed for this study, only 34 had suitable structures available [17,22,23,36,37,38,40,48,49,50,51,55,56,57,58,59,60,61,62,63,64,67,68,74,75,76,77,80,81,82,83,84,85,86,92,93,94,95,96,97,98,99,100,101,102,104,105,106,107,108,109,110,111,112]. Further availability of crystal structures is consequently important to establish a more precise structural pattern arrangement which covers all classes of HBMs/HRMs. This structural information can then be translated to machine learning algorithms which can predict HBMs/HRMs more accurately.

## Figures and Tables

**Table 1:** Origin and heme-binding parameters of the studied CP-peptides. Peptides **1**–**10** have previously been reported in a different context [5,6,8,13], but are included for reasons of comparison (**4**–**10**) and as controls (**1**–**3**).

No.	Peptide Sequence	Source	UV/vis Shift [nm]	K_D_ [µM]
**1**	AAAA**CP**AAA	control	364	3.77 ± 1.58
**2**	AAAA**C**AAAA	control	-	n.b.
**3**	AAAAAAAAA	control	-	n.b.
**4**	DESA**CP**YVM	HRI	364	2.74 ± 1.62
**5**	DESA**CP**VYM	mutant of **4**	367	9.46 ± 1.67
**6**	TPIL**CP**FHL	IRP2	368, 415	0.82 ± 0.69, 1.84 ± 1.53
**7**	SEGG**CP**LIL	IL-36α	366	2.84 ± 0.98
**8**	SSI**PC**LFYK	mutant of DGCR8	369	0.43 ± 0.35
**9**	RDQY**C**SPTK	HTS	425	n.sat.
**10**	SGGLPAPSDFK**CP**IKEEIAITSG	DP8	369	1.44 ± 0.31
**11**	TPIL**CP**HFLQPV	mutant of **12**	367 ^(≤10 µM heme)^, 416	n.sat., 0.32 ± 0.21 ^(n~2)^
**12**	TPIL**CP**F**H**LQPV	IRP2	367 ^(≤15 µM heme)^, 414	n.sat., 0.56 ± 0.44 ^(n~2)^
**13**	TPIL**CP**FLHQPV	mutant of **11**	369 ^(≤25 µM heme *)^, 414	0.71 ± 0.22 ^(n~1)^ 6.18 ± 1.12 ^(n~0.5)^
**14**	TPIL**CP**FLQHPV	mutant of **11**	368 ^(≤25 µM heme *)^, 416	0.94 ± 0.47 ^(n~0.5)^, 15.78 ± 1.74 ^(n~2)^
**15**	TPIL**CP**FLQPHV	mutant of **11**	367 ^(≤25 µM heme *)^, 418	26.93 ± 2.25 ^(n~1)^, 1.16 ± 0.90 ^(n~2.5)^
**16**	LIL**PC**GGES	mutant of **7**	416	n.p.
**17**	SEGG**PC**LIL	mutant of **7**	416	n.p.

n.b., no binding; HTS, high-throughput screening; n.sat., no saturation; n.p., no determination possible; * present, but decreasing at higher concentrations; ^n^, number of heme-binding sites.

## Data Availability

Not applicable.

## Supplementary Materials

The following supporting information can be downloaded at: https://www.mdpi.com/article/10.3390/biom13071031/s1, Table S1: List of mammalian heme-binding proteins, Table S2: Analytical characterization of peptides **11**–**17** studied herein. Figure S1: UV/vis differential spectra of heme-incubated peptide 16 (A) and peptide 17 (B).
