# Supplementary material for: Shapes and Patterns of Heme-Binding Motifs in Mammalian Heme-Binding Proteins
Source: Biomolecules. 2023 Jun 23;13(7):1031. doi: 10.3390/biom13071031 (PMC10377097; doi:10.3390/biom13071031)
Supplement: Supplementary file 1 [file biomolecules-13-01031-s001.zip › biomolecules-2454744-supplementary.pdf]

Supplementary Material

Shapes and Patterns of Heme-Binding Motifs in Mammalian Heme-Binding Proteins

Dhruv C. Rathod [1], Sonali M. Vaidya [1], Marie-T. Hopp [2], Toni Kühl [1] and Diana Imhof [1,*]

Table S1. List of mammalian transient heme-binding proteins

No.	Protein	Origin	Published heme-binding sites	PDB	Structural observation (location of motif)	pLDDT score	References
1	δ-Aminolevulinic acid synthase 1 (ALAS1)	*H. sapiens**	Cys8, Cys33, Cys108 (CP-motifs)	P13196**	All CP motifs in the loop region joining two helices	1: 70-90<br>2: 50-90<br>3: <50	[36–38]
2	δ-Aminolevulinic acid synthase 2 (ALAS2)	*H. sapiens**	Cys11, Cys38 (CP-motifs)	P22557**	All CP motifs in the loop region joining two helices	-1: 60-90<br>2: 50-70	[36,39]
3	Amyloid β	*H. sapiens**	Tyr10, His13/His14	1AMB	All motifs in centre of α-helices	-	[40–44]
4	Arginyl-Transferase (ATE1)	*M. musculus*	Cys411 (CP-motif)	O95260**	In the loop region joining helices and β-sheets	80-100	[45]
5	Arginyl-tRNA-Synthetase (ArgRS)	*H. sapiens*	Cys115	4Q2T/1F7U	In the β-sheet just before α-helix starts	-	[46]
6	Bach1	*M. musculus*	Cys438, Cys464, Cys495, Cys649 (CP-motifs)	O14867**	1-3 : In the loop, 4: In loop region joining two helices	1-3: < 50,<br>4: 90-70	[47–50]
7	Bach2	*H. sapiens*	Cys369, Cys499, Cys506 (CP-motifs)	A0A7L0PRE8**	All in loop region	<50	[51–53]
8	Big Potassium (BK) channel	*H. sapiens*	Cys612/Cys615/His616 (CXXCH-motif)	6ND0	In the loop joining two helices	-	[17,54–57]
9	Complement component 1q (C1q)	*H. sapiens*	not specified	-	-	-	[58–60]
10	Complement component 3 (C3)	*H. sapiens*	not specified	-	-	-	[59,60]
11	Circadian locomoter output cycles protein kaput (CLOCK)	*M. musculus*	His144, Cys195	6QPJ	1: In the loop region, 2: part of β-sheet	-	[61]
12	Cystathionine-β-synthase (CBS)	*H. sapiens*	Cys15 (CP-motif), Cys52, His65	1JBQ	HBMs not modelled	-	[62–65]
13	DiGeorge critical region 8 (DGCR8)	*H. sapiens*	Cys352	6LXD<br>(Seq not modelled)	HBMs not modelled	-	[35,66]
14	Dipeptidyl peptidase 8 (DPP8)	*H. sapiens*	Cys506 (CP-motif)	-	In the loop between two β-sheets	-	[6]
15	Eukaryotic translation initiation factor 2a kinase 1 (eIF2α, HRI)	*M. musculus*	Cys150 (CP-motif), Cys309	8DYS	1: In the loop region joining helices and β-sheets, 2: part of β-sheet	-	[32–34]
16	Anti-hemophilic factor (Factor VIII)	*H. sapiens*	not specified	-		-	[68,69]
17	Fibrinogen (Factor I)	*H. sapiens*	Tyr403 (suggested)	3H32	HBMs not modelled	-	[70,71]
18	Glyceraldehyde-3-phosphate dehydrogenase (GAPDH)	*H. sapiens*	not specified	-	-	-	[72–76]
19	Heme oxygenase 2 (HO2)	*H. sapiens**	His45, His256, Cys265 (CP-motif), Cys282 (CP-motif)	2Q32(H-motifs)	1: part of the helix, other motifs not modelled	-	[77–80]
20	Human period circadian protein homolog 2 (hPer2)	*H. sapiens*	Cys841, Cys962 (CP-motifs)	O15055**	All in loop regions	<50	[81]

21	Immunoglobin (IgG)	*H. sapiens*	not specified	-	-	-	[82]
22	Insulin	*H. sapiens*	His5, Tyr26 (suggested)	1UZ9	HBMs not modelled	-	[83]
23	Interleukin-36 (IL36) α,β,γ	*H. sapiens*	Cys136 (CP-motif), Tyr108(YH-motif)	6HPI	In the loop between two β-sheets	-	[20]
24	Iron regulatory protein 1 (IRP1)	*H. sapiens*	Cys118, Cys300 (CP-motifs)	Q9HBB2**	1 : In loop region joining α-helix and β-sheets<br>2 : Last residue of the α-helix before loop starts	> 90	[29–31]
25	Iron regulatory protein 2 (IRP2)	*H. sapiens*	Cys201 (CP-motif), His204, Cys375 (CP-motif)	6VCD	In loop between two helices (CP motifs)<br>(motif1 and 2 not modelled)	-	[29–31]
26	Janus kinase 2 (JAK2)	*H. sapiens*	His974, Cys1092 (CP-motif)	7UYW	In loop between two helices (CP motifs)	-	[85]
27	K_{ATP} channels	*R. norvegicus*	Cys629/His631/Tyr648/His649	7MJQ	HBMs not modelled	-	[86]
28	Neudesin	*M. musculus*	not specified	-	-	-	[87]
29	Neuferricin	*M. musculus*	not specified	-	-	-	[88]
30	Neuronal PAS domain protein 2 (NPAS2)	*H. sapiens*	His119, Cys170/His171, His335 (suggested)	Q99743**	In loop joining two helices,<br>2-3: In the middle of β-sheets<br>4: In the loop just after β-sheet ends	>90	[89]
31	NMDA Receptor	*M. musculus*	not specified	-	-	-	[90,91]
32	p53	*H. sapiens*	Cys275, Cys277 (CP-motif)	7XZZ	HBMs not modelled	-	[92,93]
33	p63	*H. sapiens*	CP-motif	3US2	-	-	[92]
34	p73	*H. sapiens*	CP-motif	-	-	-	[92]
35	PGRMC1/Sigma-2 receptor	*H. sapiens*	Tyr113	4X8Y	Part of β-sheet in loop region	-	[94,95]
36	Rev-erbα	*H. sapiens*	His602	-	-	-	[96,97]
37	Rev-erbβ	*H. sapiens*	Cys384, His568	3CQV	Cys in the helix and His in the loop	-	[97–102]
38	SOUL	*H. sapiens*	Not specified	-	-	-	[103]
39	Proto-oncogene tyrosine-protein kinase (Src)	*H. sapiens*	Cys486	2BDJ	CP motif in loop region at the interface of two helices (possible disulphide bond formation between Cys486 and Cys490)	-	[85]
40	Stanniocalcin-1 (STC 1)	*H. sapiens*	Cys114	P52823**	Last residue of the α-helix before loop starts	>90	[15]

41	Stanniocalcin-2 (STC 2)	*H. sapiens*	Cys125	8A7E	CP motif in loop region at the interface of two helices	-	[104]
42	Toll-like Receptor 4 (TLR4)	*H. sapiens*	His125, Cys169, His433, Tyr517, Cys 519	3FXI	Cys169 in α-helicle and turn region (extra-cellular domain)<br>HXH Motifs at the interface of two monomers (which forms a pocket-like region)<br>HBM with Cys519 forms hydrophobic interaction with lipid bilayer	-	[105]
43	Tryptophanyl-tRNA-Synthetase	*H. sapiens*	His130 (His129, His336, His445 assumed)	5EKD	1: part of the helix	-	[106]
44	Angiotensin-converting enzyme 2 (ACE2)	*H. sapiens*	His239, Tyr515	6M17	1,2: part of helix	-	[107]
45	Activated protein C (APC)	*H. sapiens*	His391 (HXH-motif), Tyr289 (YH-motif)	1AUT	Motif with His154 within light chain; motif with His356 located within heavy chain<br>HBM forms β-turn close to APC active site	-	[22]
46	Cluster of Differentiation 74 (CD74)	*H. sapiens*	not specified	-	-	-	[108]
47	Cortactin	*H. sapiens*	not specified	-	-	-	[4]
48	C-type lectin-like receptor 2 (CLEC2)	*H. sapiens**	not specified	-	-	-	[109,110]
49	Glycoprotein VI	*H. sapiens*	not specified	-	-	-	[110]
50	Hemoglobin (Hb)	*H. sapiens*	Cys112/His116 (CXXXH-motif), His 143/His146	1A3N	(HBMs in β-chains)	-	[23]
51	Hemopexin (Hx)	*H. sapiens*	His236 (HXH-motif), His260/His293	1QJS	1-3: part of loop region	-	[111]
52	Interleukin-1 receptor-associated kinase 1 (IRAK1)	*H. sapiens*	not specified	-	-	-	[112]
53	Kv10.1 channel (hEAG1)	*H. sapiens*	Cys541/His543/His552	-	-	-	[113]
54	Myeloid differentiation factor 2 (MD2)	*H. sapiens*	His44, Tyr61	-	HBMs located independently to LPS-binding site	-	[114]
55	Sodium channel protein type 5 (Nav1.5) channels	*H. sapiens*	not specified	-	-	-	[115]
56	Receptor for advanced glycation endproducts (RAGE)	*H. sapiens*	not specified	-	-	-	[116]
57	Tumor necrosis factor (TNFα)	*H. sapiens*	not specified	-	-	-	[108]

* In the case of these proteins, there is also further information in the literature on HBMs in other species that is not listed here. ** Structure selected from AlphaFold.

Table S2. Analytical characterization of peptides **11-17**.

No.	Peptide sequence	M_w/($M_w$calc.)[g/mo][a]	t_r*[min]	t_r**[min]*	R_f
**11**	TPILCPHFLQPV	682.34 (1361.74)[a]	80.8[c]	15.7[e]	0.57[g,] 0.71[h]
**12**	TPILCPFHLQPV	682.39 (1361.74)[a]	80.8[c]	17.3[e]	0.66[g,] 0.73[h]
**13**	TPILCPFLHQPV	682.38 (1361.74)[a]	75.1[c]	17.4[e]	0.57[g,] 0.71[h]
**14**	TPILCPFLQHPV	682.39 (1361.74)[a]	77.2[c]	17.3[e]	0.66[g,] 0.73[h]
**15**	TPILCPFLQPHV	682.39 (1361.74)[a]	77.5[c]	18.7[e]	0.66[g,] 0.88[h]
**16**	SEGGPCLIL	887.47 (886.45)[b]	44.7[d]	18.5[f]	0.51[g,] 0.69[h]
**17**	LILPCGGES	887.47 (886.45)[b]	60.2[d]	24.2[f]	0.61[g,] 0.85[h]

Peptides were measured with LC-ESI-MS and peaks were detected as a) $[M+2H]^{2+}$ and b) $[M+H]^{+}$. *Semipreparative RP-HPLC was applied for peptide purification using c) 0 – 50% eluent B (0.1% TFA in 90% acetonitrile, eluent A: 0.1% TFA in water) in 120 min and d) 10 – 60% eluent B in 120 min as gradients. **Analytical HPLC was performed using e) 20 – 60% eluent B (0.1% TFA in acetonitrile, eluent A: 0.1% TFA in water) in 40 min and f) 10 – 40% eluent B in 30 min. For TLC the following systems were applied: g) n-butanol/acetic acid/water (48:18:24, v/v) and h) pyridine/ethyl acetate/acetic acid/water (5:5:1:3, v/v).

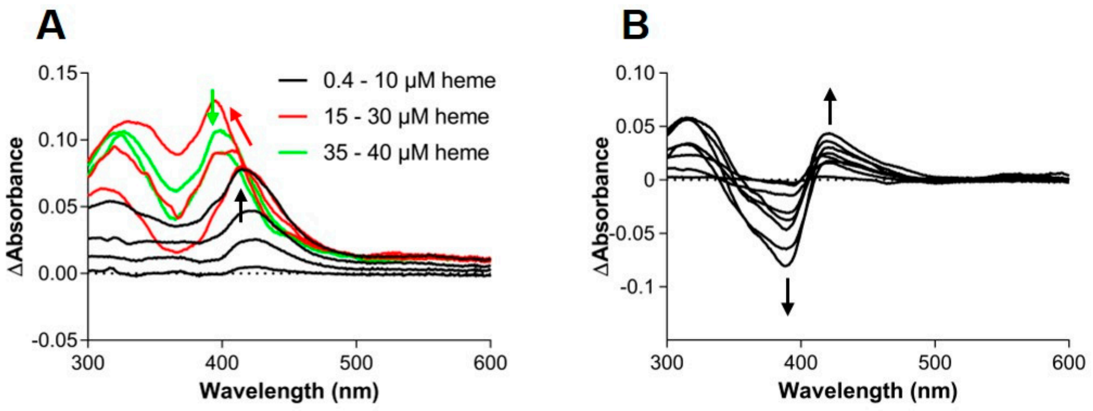

Figure S1. UV/Vis differential spectra of heme incubated with peptide **16** (A) and peptide **17** (B). Peptide **16**, the inverse sequence of **7** (resulting in a PC-motif), shows no steady spectra upon heme application but a concentration-dependent shift of the maximum from ~416 nm to ~398 nm. In contrast to the wild-type peptide **7** that depicted a shift to ~370 nm the UV/Vis data suggests a different binding mode for **16**. The spectrum of peptide **17** (PC-motif in the forward peptide sequence of **7**) deviates also from the wild-type spectrum of peptide **7**. However, a concentration-dependent trend with a maximum at ~416 nm was observed. Due to the low change of absorbance upon heme binding, KD determination was not possible.
